# Exploring the mechanisms of endophytic bacteria for suppressing early blight disease in tomato (*Solanum lycopersicum* L.)

**DOI:** 10.3389/fmicb.2023.1184343

**Published:** 2023-09-21

**Authors:** Nashwa M. A. Sallam, Heba-Alla S. AbdElfatah, Hadeel M. M. Khalil Bagy, Ameer Elfarash, Kamal A. M. Abo-Elyousr, Edward J. Sikora, Ahmed Sallam

**Affiliations:** ^1^Department of Plant Pathology, Faculty of Agriculture, Assiut University, Assiut, Egypt; ^2^Department of Genetics, Faculty of Agriculture, Assiut University, Assiut, Egypt; ^3^Department of Arid Land Agriculture, Faculty of Meteorology, Environment and Arid Land Agriculture, King Abdulaziz University, Jeddah, Saudi Arabia; ^4^Department of Entomology and Plant Pathology, Auburn University, Auburn, AL, United States; ^5^Department Genebank, Leibniz Institute of Plant Genetics and Crop Plant Research (IPK), Gatersleben, Germany

**Keywords:** biocontrol agents, 16S-rDNA, gene expression, GC-MS analysis, *Alternaria solani*

## Abstract

Controlling early blight of tomatoes using endophytic bacteria is an eco-friendly and sustainable approach to manage this common fungal disease caused by *Alternaria solani, Alternaria alternata*, and *Curvularia lunata*. Endophytic bacteria are microorganisms that live inside plant tissues without causing harm and can help protect the host plant from pathogens. In this work, twenty endophytic bacterial isolates from tomato healthy plants were tested against pathogenic fungal isolates that caused early blight disease *in vitro*. Out of the 20 tested isolates, three (B4, B7, and B17) were considered effective isolates against the growth of fungal pathogens. The three isolates were recognized as *Enterobacter cloacae* HS-6 (B4), *Pseudomonas gessardii* HS-5 (B 7), and *Pseudomonas mediterranea* HS-4 (B17) using 16s-rDNA sequencing. Different concentrations of bacterial cultural diltrates at 20, 40, and 60% were tested for their antagonistic effects on the development of pathogenic fungi *in vitro*. The lowest dry weights of pathogenic isolates in all bacterial culture filtrates were discovered at 60%. In all culture filtrates, phenolic compounds showed the largest peak area. Under greenhouse conditions, the least disease severity of tomato early blight was found for E. cloacae and its culture filtrate compared to other treatments. Real-time PCR was used to examine the expression pattern of the defense response gene β-1.3 glucanase gene in infected tomato plants with pathogenic fungi (control) as well as its relations with efficient biocontrol agent (*E. cloacae*). The expression of the gene increased substantially and significantly after three days from the inoculation-infected plants with *C. lunata* and *E. cloacae* while it reached the maximum after five days from the inoculation with *A. alternata*, *A. solani* and *E. cloacae*. Our study concluded that the endophytic bacterial isolate *E. cloacae* can be considered a promising biocontrol agent for preventing tomato early blight.

## Introduction

Tomatoes are one of the main fruits eaten as vegetables around the world, with production of 189 tons worldwide in 2021 (FAOSTAT, 2022). Early blight (EB) is a destructive disease of tomato and occurs wherever tomatoes are grown (Sallam, 2011). The disease is particularly problematic in regions with warm, humid climates, but it can also occur in cooler environments (Nashwa and Abo-Elyousr, 2012). EB is caused by several species of *Alternaria* (*solani, tomatophila, alternata*) and *Curvularia lunata* (Iftikhar et al., 2016; Adhikari et al., 2017; AbdElfatah et al., 2021). As the fungus grows within the plant tissues, it causes characteristic symptoms, including the formation of small, circular to irregularly shaped lesions on the leaves, stems, and fruit. These lesions start as small, water-soaked spots that gradually enlarge and turn dark brown or black. Management of EB often relies on the repeated use of fungicides during the growing season. However, limited use of synthetic fungicides would be preferred in a sustainable system in accordance with global trends toward environmentally friendly disease management (Nashwa and Abo-Elyousr, 2012; Adhikari et al., 2017; Jabeen et al., 2021). An alternative approach for controlling EB might be the use of biological control agents (BCAs; Czajkowski et al., 2011). Endophytic bacteria (i.e., bacteria that successfully colonize the interior plant tissues) can play an important role in the management of plant diseases (Lanna-Filho et al., 2017; Sharf et al., 2021). Endophytic bacteria have the ability to adapt to harsh environmental conditions and could potentially be used as an alternative to synthetic fungicides (Gómez-Lama Cabanás et al., 2014; Eljounaidi et al., 2016; Larran et al., 2016). A wide variety of endophytic bacteria have been detected in tomato (Yang et al., 2020). The most common genera found in the roots, stems, and leaves of tomato plants were from the genera *Acinetobacter*, *Enterobacter*, and *Pseudomonas* (Yang et al., 2020). Various mechanisms were reported for their biological control potential including inhibition of pathogen growth by digestive enzymes, surface-active compounds (antibiosis), antibiotics, and toxins (Obeid et al., 2017; Khan and Javaid, 2021; Sharf et al., 2021; Esmail et al., 2023). Therefore, additional studies using gas chromatography and mass spectrophotometry analysis (GC-MS) are needed to determine the chemical profile of bacterial antagonists’ active compounds. These methods can be used to identify bioactive components in bacterial metabolites for the creation of novel antibacterial substances for use in sustainable agriculture (Hameed et al., 2013).

The possible mechanism for the synergistic effect of bacterial endophytes is the enhancement of the expression level of pathogeneses-related protein (PR) during disease progression (Sharifi and Ryu, 2016). Such PR proteins involve many enzymes such as chitinases and *β*-*1*,3-glucanases which lyse the attacking cells and enhance the cell wall boundaries. This provides resistance against infection and cell death by plant pathogens. The expression analysis of PR gene can be investigated by RT-PCR (Sallam et al., 2019).

Our study was developed to look at endophytic bacteria as an alternative to synthetic fungicides for management of EB. The objectives of this study were to (a) isolate different strains of endophytic bacteria, (b) investigate the antagonistic capability of endophytic bacteria against isolates of EB *in vitro* identify and classify these isolates using 16s-rDNA and evaluate the activity of endophytic bacterial culture filtrate against the growth of isolates of EB *in vitro* on tomato leaves.

## Materials and methods

### Isolation of endophytes

Roots, leaves, and stems of disease-free greenhouse-grown tomato plants were used to collect isolates of endophytic bacteria from their internal tissues as described by Abdel-Hafez et al. (2015). Tomato tissues were subsequently cleaned using tap water. Tissues were divided into relatively small pieces and sterilized by dipping in a solution of 5% sodium hypochlorite for 10 min followed by dipping in 70% ethanol for 5 mins. The samples were then rinsed with distilled water three times and dried in sterilized filter paper. Afterwards, five tomato segments were arranged in Petri dishes saturated with nutrient sucrose agar (NSA). The Petri dishes were incubated at 27°C ± 1°C for 2 days. From these cultures a single bacterial colony was collected and transferred onto NSA using the streak plate technique to obtain a pure bacterial colony (Ziaei-Nejad et al., 2006). The NSA consisted of beef extract (3.0 g), peptone (5.0 g), agar (15 g), sucrose (5.0 g), and distilled water (1,000 mL).

### Source of pathogenic isolates

The pathogenicity of pathogenic isolates (*C. lunata, A. alternata*, and *A. solani*) had been examined by AbdElfatah et al. (2021) and it shown significant virulence.

### Antagonistic capability of endophytic bacteria *in vitro*

Twenty endophytic bacterial isolates were tested *in vitro* for antagonistic effectiveness against *C. lunata, A. alternata*, and *A. solani*. A seven-day-old mycelial disc (5 mm in diameter) of each pathogen was placed in the middle of a PDA medium plate, and endophytic bacteria strains were streaked on both sides of the plate with a sterile inoculation needle (Hyder et al., 2020). Inoculated plates with each pathogen alone were used as controls. Each treatment was replicated three times. The plates were incubated at 27°C ± 1°C for 7 days. The inhibitory effects of endophytic bacterial isolates on the linear growth (cm) of the pathogen were determined using the following formula (Sallam et al., 2019): inhibition growth = control growth (cm) − growth in treatment (cm).

### Molecular identification of endophytic bacteria by using 16s-rDNA

Three endophytic bacterial isolates (B4, B7, and B17) that exhibited the greatest suppression against *C. lunata, A. alternata*, and *A. solani* were molecularly identified using16S-rDNA gene sequences. In sterile test tubes with 10 mL of NSB, bacterial isolates were cultured and then incubated at 27°C ± 1°C for 48 h (Zimbro et al., 2009). DNA was extracted using pathogens-spin DNA/RNA extraction kit (Intron Biotechnology Company, Korea). DNA samples were shipped for sequencing at the SolGent Company (Daejeon, South Korea). The polymerase chain reaction (PCR) was conducted using two universal primers 27F (5′-AGAGTTTGATCCTGGCTCAG-3′) and 1492R (5′-GGTTACCTTGTTACGACTT-3′). A size nucleotide marker (100 base pairs) was used to determine the PCR products. Sequences were also blasted in the National Center of Biotechnology Information (NCBI) website. Phylogenetic analysis of sequences was performed using MegAlign (DNA Star) software version 5.05.

### Screening activity of endophytic bacterial culture filtrate *in vitro*

The bacterial isolates B4, B7, and B17 [*E. cloacae* (Ec)*, P. gessardii* (Pg), and *P. mediterranea* (Pm)] were inoculated into Erlenmeyer flasks (1,000 mL) containing 500 mL of sterile NSB and kept on a rotary shaker at 150 rpm at 27°C ± 1°C for 48 h. The bacterial cell suspension was centrifuged for 8 min. at 10,000 g to obtain a pellet of each culture filtrate (CF). The CF was sterilized in the Zeits filter and kept in a sterile flask (500 mL) before being used (Fischer et al., 2006).

A measured amount of CF-Ec, CF-Pg, and CF-Pm were added individually into sterilized Erlenmeyer flasks containing PDB medium (100 mL) to obtained concentrations at 20%, 40%, and 60% using a sterile pipette. Instead of CF, water was used in PDB as a control. Each flask containing modified media was inoculated with one disc of each pathogen (5 mm) and incubated at 27°C ± 1°C. After 7 days of incubation (the growth of pathogenic fungi was completed in control), the fungal mycelium was filtered from the culture liquid using Whatman (No. 1) filter paper. The mycelial pellet was washed in distilled water before being dried overnight at 70°C. Subsequently, the dry weight of the fungus was measured (Srivastava A. K. et al., 2010).

### GC-MS analysis of the endophytic bacteria culture filtrate

Detection of active bio-molecules in the crude extract of CF-Ec, CF-Pg, and CF-Pm was carried out through GC-MS (GC Trace 1300 Thermo Scientific) in the Department of Chemistry, Faculty of Science, Assiut University. To identify volatile compounds, GC-MS was performed using capillary column TG-5MS, 30 m × 0.25 mm × 1 μm equipped with GC Trace 1300 Thermo Scientific. A single quadruple mass spectrometer (ISQ 7000 Thermo Scientific) was utilized. The carrier (He, 99.999%) flow was 1 mL min^−1^, split 10:1, and injected at volumes 2 μL. The column temperature was initially kept at 110°C for 5 min. The temperature was increased to 200°C for 5 min at a rate of 10°C/min. The temperature was further increased to 250°C at a rate of 5°C/min for 5 min. The injector temperature during the analysis was 250°C. The electron impact energy was 70 eV and the ion source temperature was set at 250°C. Electron impact (EI) mass scan (m/s) was recorded in the 40–650 amu range (Kumar et al., 2008).

### Evaluation of endophytic bacteria and culture filtrate on tomato early blight under greenhouse conditions

#### Plant material

All experiments were carried out under greenhouse conditions at the Department of Plant Pathology, Faculty of Agriculture, Assiut University. Autoclaved sand clay soil (3 kg/ pot) was filled into sterilized plastic pots (30 cm diameter). Thirty-day old tomato seedlings (cv. 844) were transferred into pots (two seedlings/pot). The plants were fertilized with 10 g slow-release fertilizer per kg (NPK 12, 4, 6). The pots were kept at a relative humidity (RH) of 68%–80% and temperature of 30°C ± 5°C.

#### The spore suspension of pathogenic isolates

Fungal isolates were grown on plates containing PDA and incubated for 15 days at 27°C ± 1°C. Conidial isolates were gently rubbed by adding 10 mL of the distilled water to each plate using a sterile needle. The spore suspension of each isolate was adjusted to 4 × 10^6^ CFU/mL. Thirty days after planting, tomato plants were sprayed with a spore concentration of each isolate [infected plants (IP)] using an atomizer or with a water control (healthy plants; Kumar et al., 2017). Polyethylene bags were used to cover the plants for 48 h. Each treatment contained three replicates (three pots/replicate).

#### Bacterial cell suspension and culture filtrate

A single colony of Ec, Pg, and Pm was grown in a 1,000 mL Erlenmeyer flask containing NSB (750 mL) and incubated for 48 h at 27°C ± 1°C on a rotary shaker at 150 rpm. The suspension of bacterial cells was then centrifuged at 10,000 g for 8 min. The cells were suspended in sterile distilled water and adjusted to 5 × 10^8^ CFU/mL using a spectrophotometer at a wavelength of 620 nm (Mc-Guire and Kelman Gomez and Gomez, 1984). Each CF was prepared at 60% in Erlenmeyer flasks (1,000 mL) as described previously.

Three days after inoculation, plants were divided into two groups. The first group was sprayed with each bacterial cell suspension while the second group was sprayed with each bacterial culture filtrate. Only water was sprayed on the control plants. All plants were covered with a polythene bag for 48 h. After 30 days, disease severity was recorded using a visual scale of 0 to 4 (Sallam, 2011; Zheng et al., 2015) with 0 = no spots on leaves, 1 = spots occupied < 25% of leaf area, 2 = spots occupied between 26% to 50% of leaf area, 3 = spots occupied between 51% to 75% of leaf area and, 4 = spots occupied between 76 to 100% of leaf area. The following formula was used to determine:

Disease severity (%) = Σ (n **×** r)/NR **×** 100 with *n* = number of infected leaves per plant, r = numerical rate of infected leaves, *N* = total number of leaves per plant and R = maximum numeric rate. The microorganism was re-isolated from the inoculated plants and symptoms of EB were observed after following Koch Postulates.

#### Expression analysis of the PR gene in tomato leaves

##### Gene expression analysis

The endophytic bacteria Ec was used in the gene expression experiment as it had the lowest disease severity value for EB in the previous experiment. The expression gene of *β*-*1*,3-glucanase was examined 3, 5 and 10 days after inoculating plants with EC for each treatment. Plants that were not inoculated served as a healthy control. Plants inoculated with only one fungal pathogen were used as infected controls. This experiment included a healthy control, an infected control, and three treatments with three different fungi.

In this experiment, leaves of plants samples (0.1 g) were collected 3, 5, and 10 days after inoculation with bacterial cell (three replicates/treatment). Additionally, leaves from the healthy and infected control plants were included to compare the gene expression pattern among the different treatments. All leaves were transferred to liquid N and stored immediately at −80°C. The RNA was extracted using the RNeasy Plant Mini Kit (Qiagen, Valencia, CA, United States). All samples were treated with DNase I, RNase-free (Thermo Fisher Scientific, United States) to obtain purified RNA, Complementary DNA (cDNA) was prepared by the Revert Aid First Strand cDNA Synthesis Kit protocol (Thermo Fisher Scientific, United States). Finally, all samples were prepared for real-time PCR using Maxima SYBR Green/ROX qPCR Master Mix (2X; Thermo Fisher Scientific, United States). Thermocycling was performed using the following conditions: 2 min at 50°C, 10 min at 95°C, and 40 cycles alternating between 15 s at 95°C and 1 min at 60°C. The gene expression levels for each sample were estimated on three analytical replicates and analyzed using the data dCt method (Silver et al., 2008). The analysis of variance (ANOVA) was calculated to test the significant differences among replications and treatments by PLABSTAT software (Utz, 1997). The repeatability of treatment was calculated using PLABSTAT software (Utz, 1997).

##### Effect of endophytic bacteria and culture filtrate on tomato early blight disease under field conditions

The effect of Ec, Pg, Pm, CF-Ec, CF-Pg, and CF-Pm on EB severity was carried out at the Experimental Farm of Plant Pathology Department, Faculty of Agriculture, Assiut University, Assiut, Egypt in 2017/2018 and 2018/2019 growing seasons. Field plots (3 × 3.5 m) included two rows (every 3 m in length and 5plants/row) arranged in a completely randomized block design with three replications. Tomato plants (cv. 844) at 30 days old were transplanted in field. Spraying tomato plants with a spore concentration of pathogenic isolates (4 × 10^6^ CFU/mL) during flowering time. Following 3 days of pathogen inoculation, plants were treated with antagonistic bacteria (5 × 10^8^ CFU/ml) or their culture filtrates (60%). The control group consisted of infected plants that were sprayed with water. Three replicates were used in each treatment (one plot/ rep.). One month later, disease severity was assayed as previously described.

### Statistical analysis

The statistical analyses was a two-way ANOVA using SPSS 19.0 software. Means were measured by multiple range tests performed by Duncan and statistical significance was assessed at a level of 5% (Gomez and Gomez, 1984).

## Results

### Antagonistic capability of endophytic bacteria *in vitro*

Twenty isolates of endophytic bacteria isolated from root, leaves and stems of healthy tomato plants were tested *in vitro* against the growth of pathogenic isolates (*A. solani*, *A. alternata*, and *C. lunata*). All bacterial isolates inhibited growth (Supplementary Figure S1)of the fungal pathogens at different degrees (cm) compared with the control (Table 1). Isolates B1, B4, B7, B8, B10, B14, B15, B17, and B18 showed the highest inhibition of growth of the fungal pathogens whereas isolates B2, B5, B6, B9, B10, B11, B12, B19, and B20 had a moderate effect on mycelial growth. Isolates B13 and B16 had the least effect on pathogen development.

**Table 1:** Antagonistic capability of endophytic bacteria *in vitro*.

Isolates of endophytic bacterial (B)	Inhibition growth of fungal pathogens (cm)					
	*Alternaria solani*		*Alternaria alternata*		*Curvularia lunata*	
1	4.67	h~m	6.00	b~f	3.05	q~u
2	5.00	f~k	6.50	bcd	3.55	m~u
3	4.33	i~p	5.33	e~i	6.00	b~f
4	2.67	tu	2.93	r~u	2.57	u
5	4.50	h~o	6.17	b~e	4.00	j~r
6	5.50	d~h	7.00	ab	3.42	o~u
7	3.17	q~u	3.97	j~r	2.72	stu
8	4.42	h~p	5.33	e~i	3.72	l~t
9	4.33	i~p	6.50	bcd	4.20	i~q
10	5.33	e~i	4.83	g~l	3.88	k~r
11	5.83	c~g	6.00	b~f	4.02	j~r
12	6.67	bc	5.33	e~i	3.50	n~u
13	6.83	bc	6.83	bc	4.00	j~r
14	4.83	g~l	4.83	g~l	3.83	l~s
15	4.50	h~o	6.17	b~e	3.33	p~u
16	6.50	bcd	6.17	b~e	3.83	l~s
17	4.67	h~m	4.33	i~p	3.42	o~u
18	4.00	j~r	4.63	h~n	3.72	l~t
19	4.83	g~l	5.83	c~g	3.88	k~r
20	5.50	d~h	5.10	e~j	4.02	j~r
Control	0.00	a	0.00	a	0.00	a
Mean	5.05	B	5.69	a	3.93	c

LSD_0.05._ Means of three replicates in a column designated by the same letter(s) are not significantly different according to Duncan’s multiple range test (*p* = 0.05).

### Molecular identification of endophytic bacteria by using 16s-rRNA

**Figure 1:**
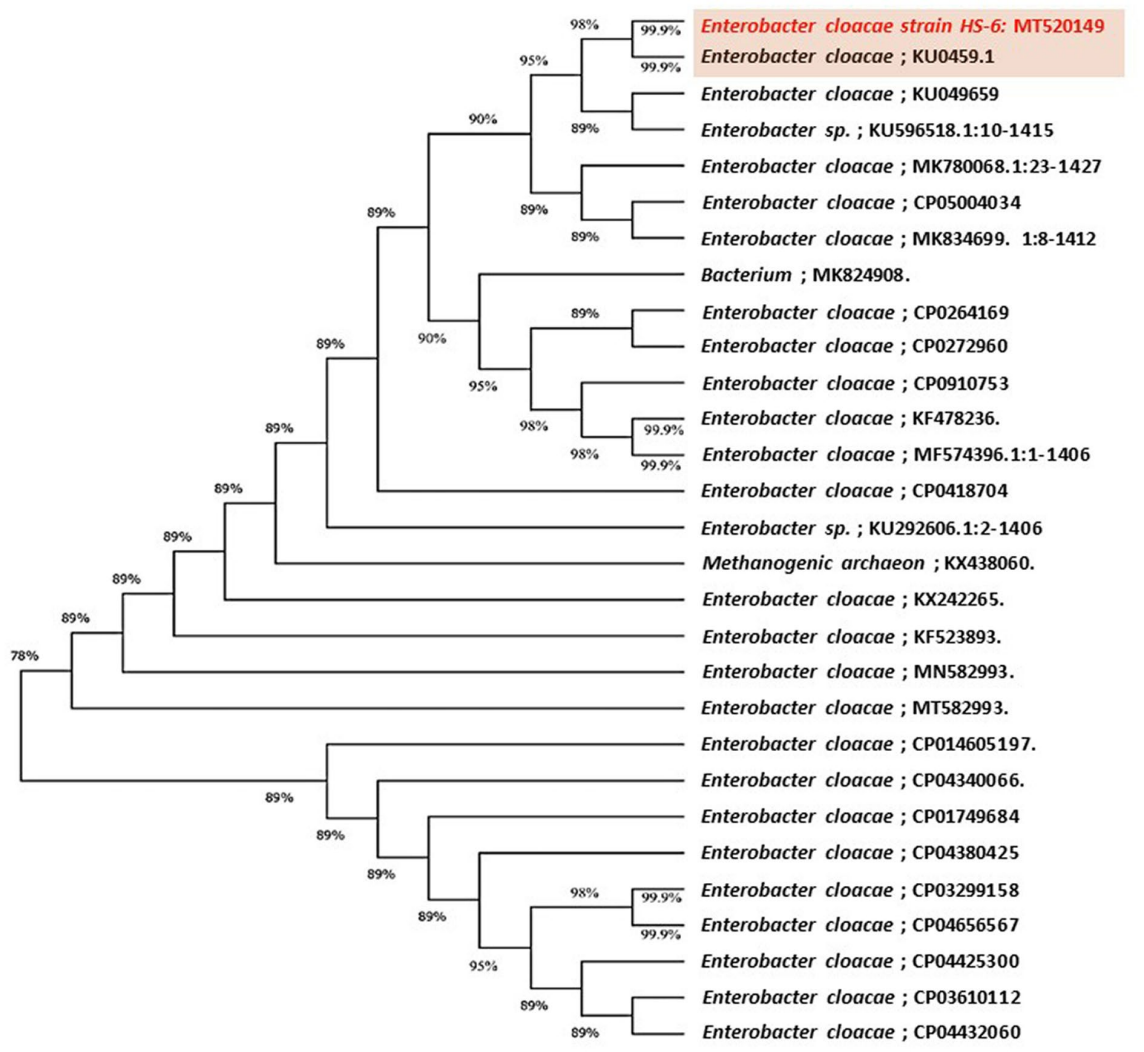
Maximum likelihood phylogenetic tree-based ribosomal region 16s-rDNA and bootstrap support values > 60 (BS) are given at the nodes (BS) on 16s-rDNA sequences of rDNA of the isolated bacteria strain (No. 4) in the present study was *Enterobacter cloacae* MT520149 aligned with closely related sequences accessed from the Gen Bank. The marker reflects the relative phylogenetic distance measurement. Red species refer to the isolated ones detected in this study.

**Figure 2:**
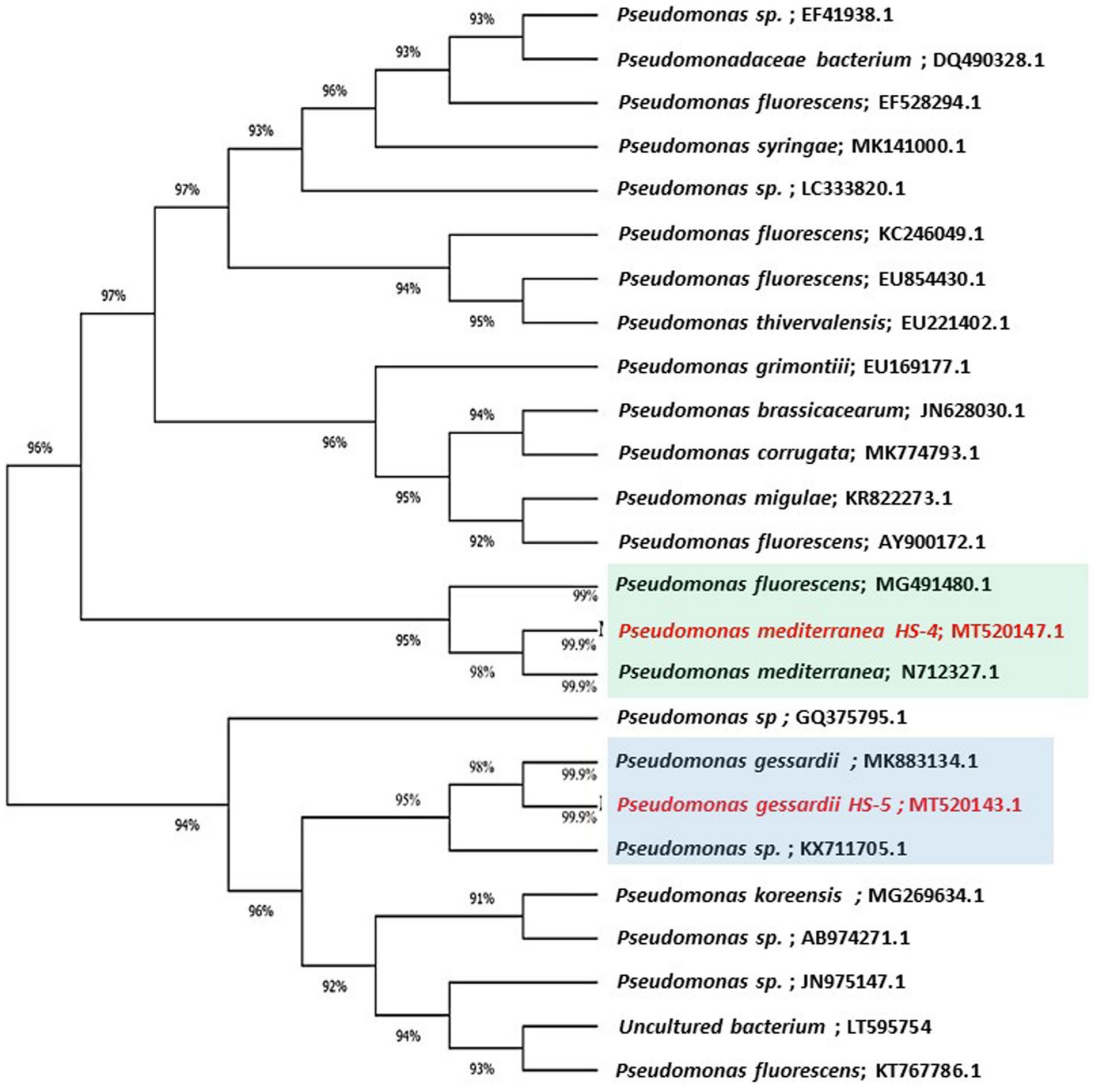
Maximum likelihood phylogenetic tree-based ribosomal region 16s-rDNA and bootstrap support values > 60 (BS) are given at the nodes (BS).16s-rDNA sequences of rDNA of the isolated bacteria strains (No. 7 and 17) in the present study were *Pseudomonas gessardii* MT520143.1 and *Pseudomonas mediterranea* MT520143.1, respectively. Which were aligned with closely related sequences accessed from GenBank. The marker reflects the relative phylogenetic distance measurement. Red species refer to the isolated detected in this study.

The highly antagonistic bacterial isolates B4, B7, and B17 were identified using the ribosomal region 16s-rDNA based on preliminary tests for the antagonistic capability of endophytic bacteria against pathogens *in vitro* studies. Identification of isolated bacteria was carried out based on the morphological characteristics and molecular identification. The resulting sequences were compared in the NBCI database using the BLAST program. The sequence analysis of the B4 isolate (1,406 bp) revealed a 99% match to *Enterobacter cloacae* strains KU049659.1 which was designated as *Enterobacter cloacae* HS-6 on the phylogenetic tree (Figure 1). Under the accession number MT444991, the sequence was deposited in GenBank. Isolate B7 generated the ribosomal DNA gene with a band size of 1,402 bp as shown in the phylogenetic tree (Figure 2). BLAST analysis showed that the sequence of isolate B7 was 99% identical to *Pseudomonas gessardii* strains MK883134.1. *P. gessardii* HS-5 was entered with the accession number MT520143 in GenBank. The sequence data of the 1,413 bp fragment was formed by isolate B 17. The sequence alignment data and the established phylogenetic tree showed that the isolate *Pseudomonas mediterranea* HS-4 has a 100% resemblance to *P. mediterranea* strain MN712327 and was registered in GenBank with an accession number MT520147 (Figure 2).

### Screening activity of endophytic bacterial culture filtrate *in vitro*

**Figure 3:**
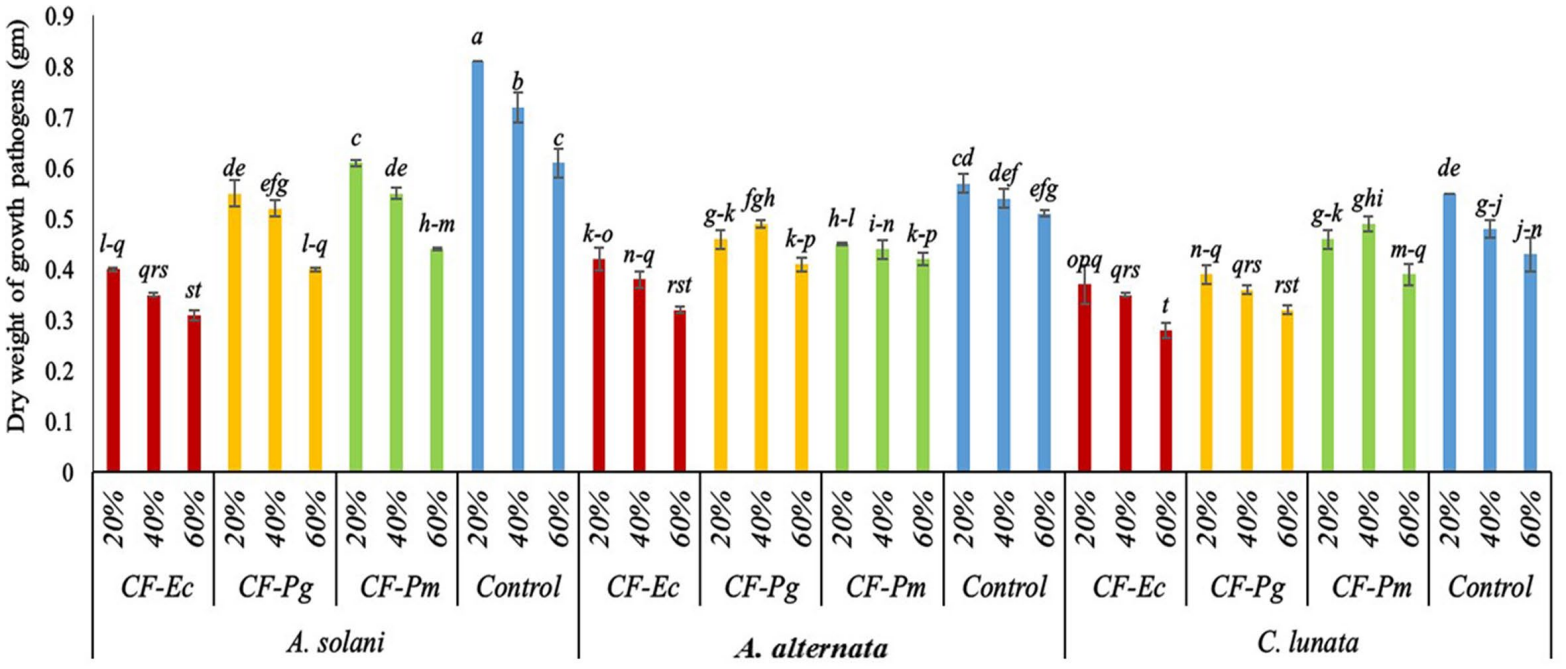
Effect of bacterial culture filtrate on the dry weight of pathogens growth (*Alternaria solani*, *Alternaria alternata*, and *Curvularia lunata*) by using three conc. (20%, 40%, and 60%). Each point in the histogram represents a mean value of three replicates and the vertical bars indicate + SE which are significantly different at *p* < 0.05.

The activity of *CF* of endophytic bacterial isolates was tested against the growth of *A. solani*, *A. alternata*, and *C. lunata in vitro*. Results indicate that the various concentrations of bacterial filtrate significantly decreased the dry weight of pathogenic fungi compared to the control (Figure 3). All CF at 60% concentration showed the lowest dry weight of pathogenic isolates followed by the 40% and 20% CF. There were significant variations among CF-Ec, CF-Pg, and CF-Pm in this experiment with CF-Ec producing the lowest dry weight of pathogenic isolates followed by CF-Pg and CF-Pm.

### Analysis of antagonistic bacterial culture filtrate using GC-MS

Based on the effect of CF-Ec, CF-Pg, and CF-Pm on the dry weight of fungal growth, the analysis of the filtrate components was studied using GC/MS. The 25 volatile organic compounds (VOCs) produced by the ethanol extract of CF-Ec were identified (Supplementary Table 1). The highest peak was at 45.27% with 22.60% recognized as Phenol, 2,4-di-tert-butyl-or Phenol, 2,4-bis(1,1-dimethylethyl)- and 2-Butanone, 3-hydroxy- (ç-Hydroxy-á-oxobutane), respectively. Other substance, such 2,3-Butanediol, had peak areas of about 8.60%. The remainder of the compounds had a small peak area ranging from 1% to 2%. However, CF-Pg had 20 VOCs (Supplementary Table 2). The peak area of phenol, 2,4-bis(1,1-dimethylethyl)- or phenol, 2,4-di-tert-butyl, was at 34.49%; subsequent peak areas for 1-Hexadecanol or n-Cetyl alcohol at 9.55%, 1-Nonadecene at 8.92%, 1-Hexadecanol (n-Cetyl alcohol) at 7.58%, and 3,5-Dimethyl-2-hexene at 7.32%. The remaining substances exhibited a minimum peak area ranging from 1 to 6%. There were a total of 20 VOCs identified in the CF-Pm analyses. The peak area for phenol, 2,4-bis (1,1-dimethylethyl), or phenol was 35.59%. Compounds such as 1-Docosene, 1-Hexadecanol (n-Cetyl alcohol), and 2-Hexene, 3,5-dimethyl- exhibited peak areas of 9.50, 8.96%, 7.25%, and 7.03%, respectively (Supplementary Table 3). Other compounds ranged from 1% to 5% and had very modest peak areas.

### Evaluation of endophytic bacteria and culture filtrate on early blight of tomato under greenhouse conditions

**Figure 4:**
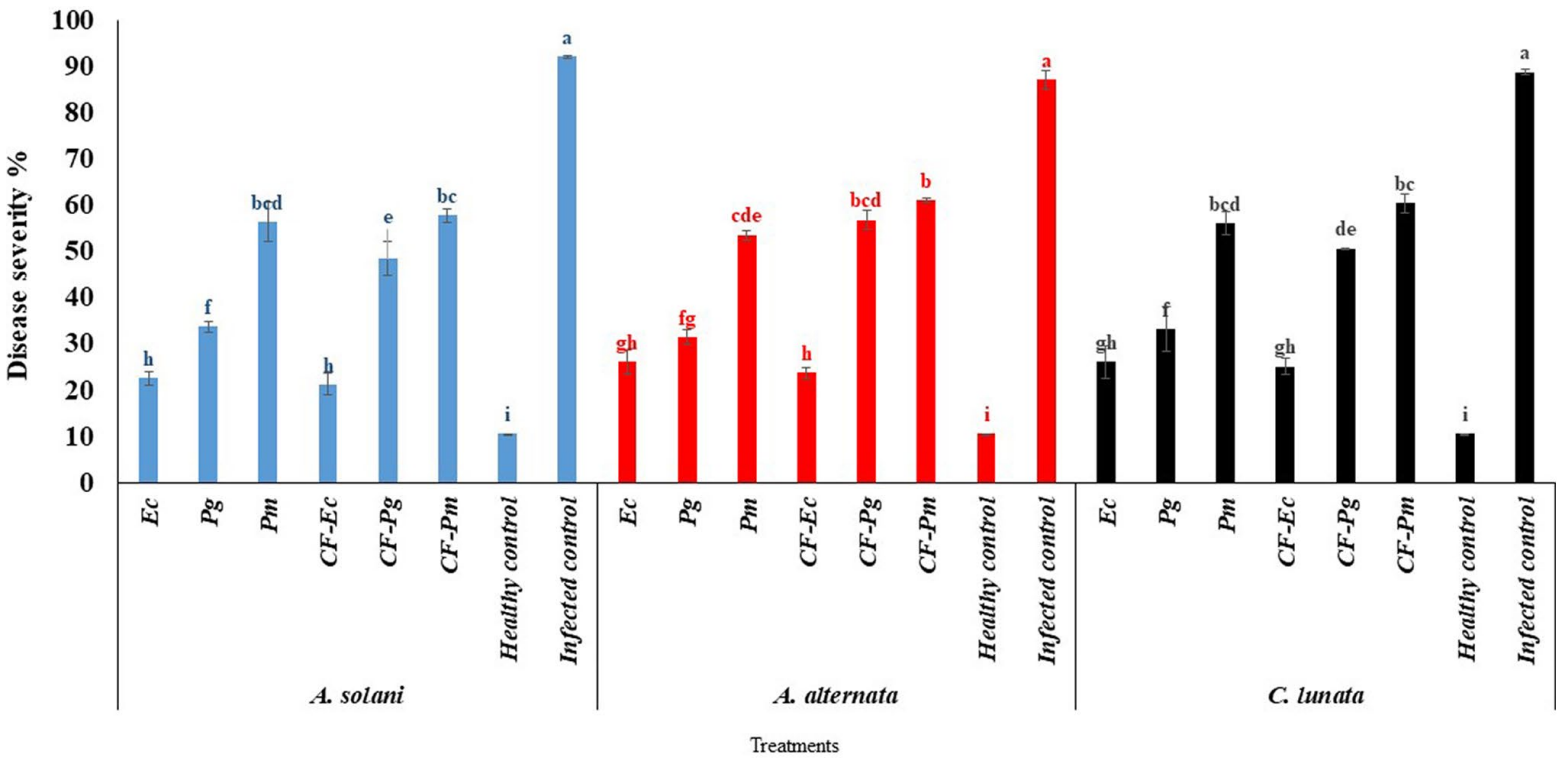
The effect of antagonistic bacteria and their culture filtrate on early blight disease on tomato causal agents (*Alternaria solani*, *Alternaria alternata*, and *Curvularia lunata*) under greenhouse conditions. Each point in the histogram represents a mean value of three replicates and the vertical bars indicate + SE which are significantly different at *p* < 0.05.

The endophytic bacterial isolates Ec, Pg, and Pm and their cultural filtrate at 60% (CF-Ec, CF-Pg, and CF-Pm, respectively) were tested on the incidence of EB on tomato under greenhouse conditions. Infected tomato plants treated with each antagonistic bacteria or their CF significantly reduced EB severity compared with the control (Figure 4). The CF-Ec significantly reduced the severity of EB compared to the other two bacterial filtrates. CF-Pg had a moderate effect on EB severity and CF-Pm had the least effect. Plants treated with Ec or CF-Ec reduced symptoms of EB compared to the other treatments.

### Expression analysis of the PR gene in tomato leaves revealed by RT-PCR

**Figure 5:**
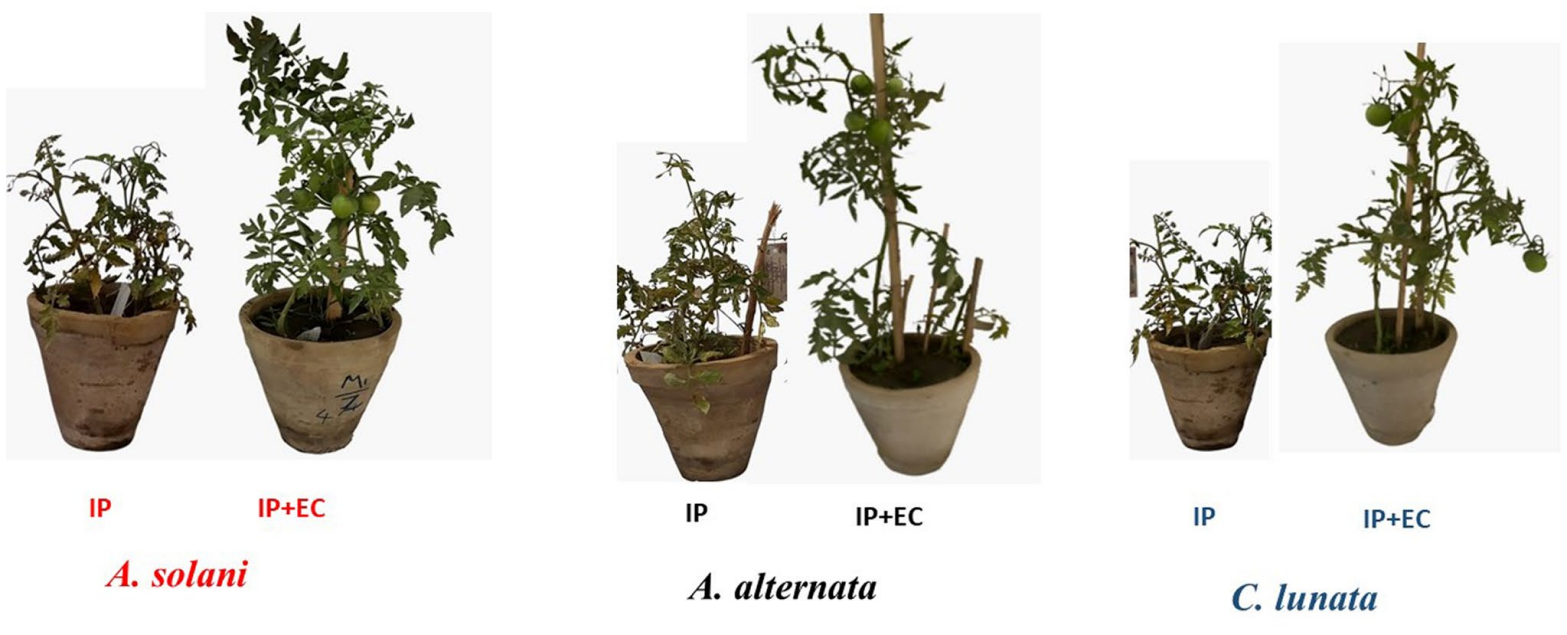
Effect of antagonistic bacterium *Enterobacter cloacae* (EC) on early blight disease of tomato. IP refers to infected plants, IP + EC refers to the infected plant by respective pathogenic fungi and treated with EC.

**Figure 6:**
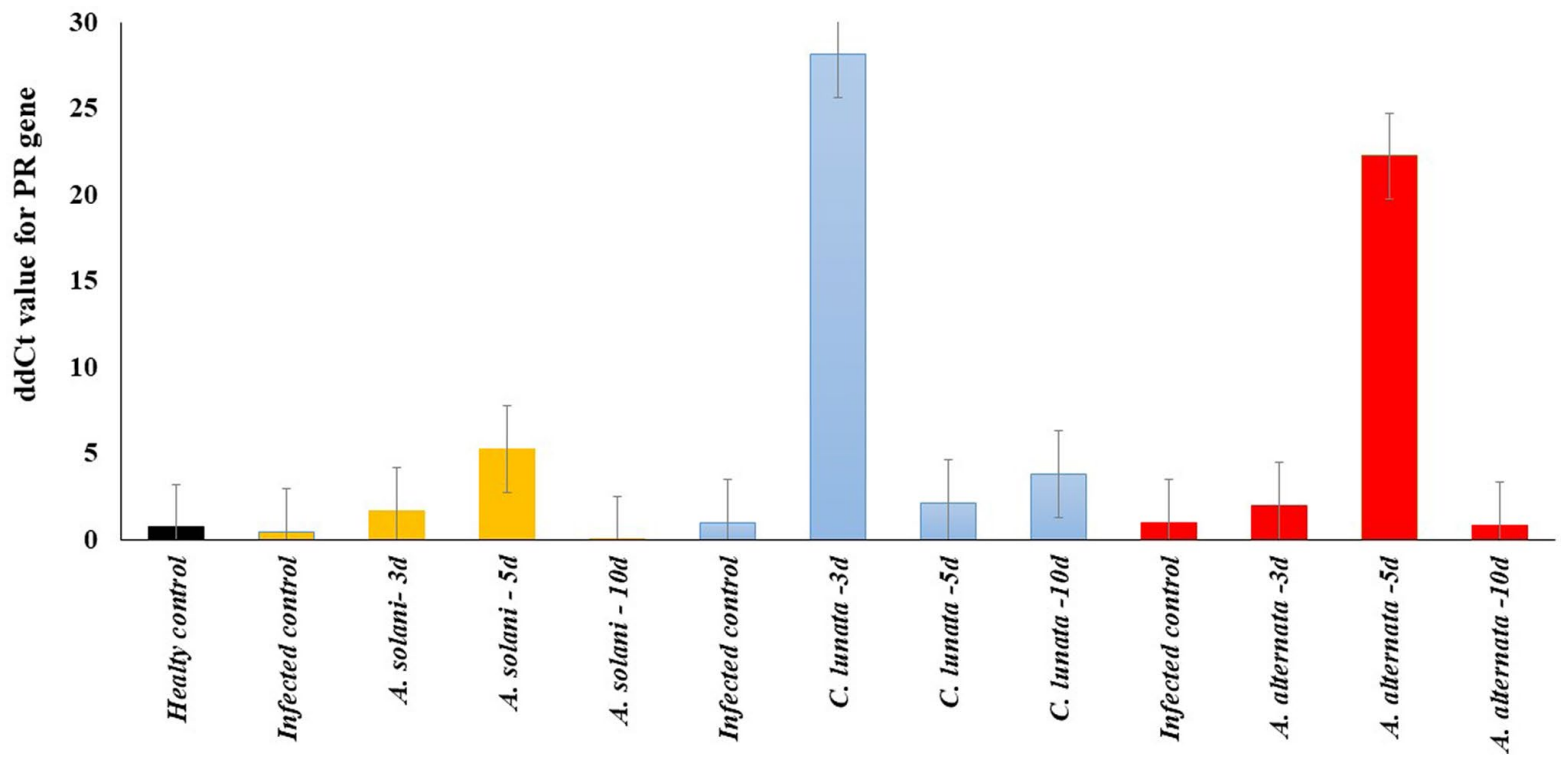
Real-time RT-PCR analysis of PR gene expression after 3, 5, and 10 days (3d, 5d, and 10d) from the treatment of the inoculated tomato leave with pathogenic fungi (*Alternaria solani*, *Alternaria alternata* and *Curvularia lunata*) and endophytic bacteria *Enterobacter cloacae* (EC).

The expression of the β-1,3*-*glucanase gene was investigated as it showed the greatest potential to reduce symptoms of EB (Figure 5). On average the expression of the gene was higher in the tomato leaves infected with *C. lunata* than the other two fungal pathogens (Figure 6).

The expression level of the defense-related β-1,3*-*glucanase gene was also tested in all treatments with Ec after 3, 5, and 10-days (Supplementary Figure S2). The expression of the β-1,3*-*glucanase gene was significantly higher in the disease control for *A. solani, C. lunata* and *A. alternata* than the healthy control. In tomato leaves infected with *A. solani*, the gene’s expression increased on the fifth day following Ec inoculation and began to decrease rapidly on the tenth day. For tomato leaves infected with *C. lunata*, gene expression reached the maximum level on the third day and declined on the fifth and tenth days. The expression of the gene in tomato leaves infected with *A. alternata* was higher on the fifth day than it was on the third and tenth days. The analysis of variance was calculated to test the differences between treatment and replications. No significant differences were found among the three replicates for each sample. Significant differences were found among all treatments with B4 (*p* > 0.01). The repeatability of gene expression across all treatments was 85%.

### Effect of endophytic bacteria and culture filtrate on tomato early blight disease under field conditions

**Figure 7:**
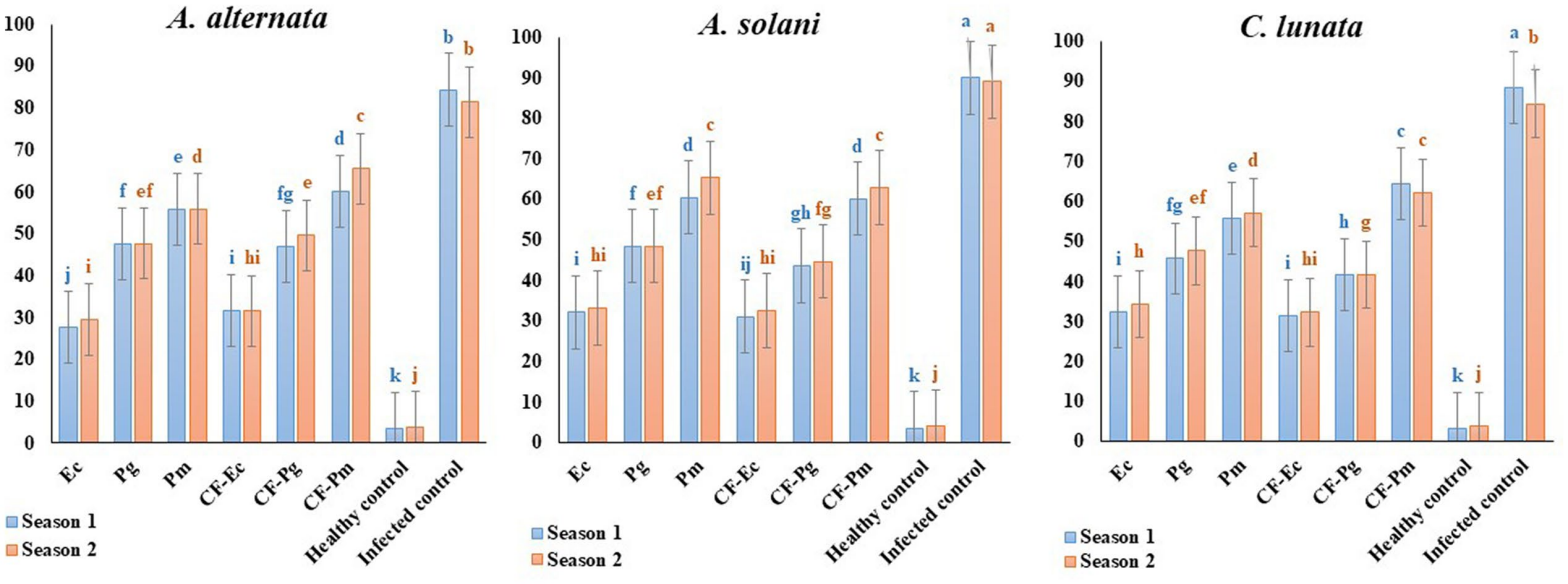
Effect of antagonistic bacteria and their *CF* on early blight disease on tomato causal agents (*Alternaria solani*, *Alternaria alternata*, and *Curvularia lunata*) under field conditions (season 1 and season 2). Each point in the histogram represents a mean value of three replicates and the vertical bars indicate + SE which are significantly different at *p* < 0.05.

The impact of antagonistic bacterial isolates (Ec, Pg and Pm) and their culture filtrates (CF-Ec, CF-Pg, and CF-Pm) on EB was assessed in the field. Spraying tomato plants infected with *A. solani*, *C. lunata* and *A. alternate* with endophytic bacteria and CF reduced EB severity compared to the control (Figure 7). Plants treated with Ec significantly reduced EB severity followed by Pg. The most severe disease was observed on plants treated with Pm. Spraying infected plants with CF-Ec showed a significant reduction of EB than CF-Pg, while CF-Pm had the least effect on EB severity in the field.

## Discussion

Bacteria that reside in plant tissues are known as endophytic bacteria. These bacteria can assist plants to fight off infectious diseases. Moreover, they may aid in stimulating plant growth (Mohamed et al., 2020; Fouda et al., 2021). In our study, 20 endophytic bacterial isolates were isolated from healthy tomato plants to determine their antagonistic potential against EB (Nakkeeran et al., 2005; Srivastava R. K. et al., 2010; Singh et al., 2014; Altinok and Erdogan, 2015). Bacterial isolates B4, B7 and B17 showed a relatively high level activity to inhibit growth of fungal pathogens *A. solani, A. alternata* and *C. lunata* in comparison to the control. This suggests that these isolates may suppress these pathogens, possibly through the production of active compounds that prevent fungal development and/or that compete with these pathogens for nutrition (Trung et al., 2022).

Using the ribosomal region 16s-rDNA, the bacterial isolates B4, B7, and B17 were identified as *Enterobacter cloacae* HS-6 (B4), *P. gessardii* HS-5 (B7) and *P. mediterranea* HS-4 (B17). The 16S-rDNA gene is often used in phylogenetic studies (Weisburg et al., 1991) because it includes hypervariable segments that provide species-specific signature sequences beneficial for bacterial identification (Altaai et al., 2014; Lo et al., 2015). Our results are consistent with those obtained from relative 16S-rDNA gene sequences indicating that this method would accurately identify *Pseudomonas* spp. and *Enterobacter* spp. Bacillus, Pseudomonas and Enterobacter were the most common genera of endophytic bacteria isolated from different healthy plant species (AlAli et al., 2022).

Our research revealed that the endophytic bacteria (Ec, Pg, and Pm) have inhibitory effects against pathogenic fungi. This may be due to the fact that these bacteria produce their own lytic enzymes and/or secondary compounds that can break down cell walls as mentioned by Grata (2016). In our study we evaluated different concentrations of bacterial filtrate (20%, 40%, and 60%) *in vitro* to determine the concentration of CF-Ec, CF-Pg, and CF-Pm that would inhibit growth of EB. In general, the best concentration for all bacterial filtrates was 60%, a percentage that would reduce fungal development. These results are similar to Sharma et al. (2021) who reported that the mycelial growth of *A. solani* was inhibited by the culture filtrate of *P. fluorescens* at a concentration of 40%. The antifungal properties of phytocompounds of *E. cloacae* and *P. fluorescens* have shown good antifungal activity against a variety of plant pathogens. Antibiotics generated from by these microorganisms were thought to be the source of antimicrobial activity (Kim et al., 2010; Wang et al., 2010).

It has been shown that endophytes constitute a dependable source of bioactive and chemically unique substances with enormous therapeutic and agricultural promise (Senthilkumar et al., 2007; Sharifi et al., 2021). Therefore, the goal of this study was to use the GC-MS technique to detect bioactive chemicals in the extracts of endophytic bacteria. CF-Ec was found to have 25 VOCs, but CF-Pg and CF-Pm only contained 20 each. Endophytes are a reliable source of bioactive and chemically unique compounds that can be used to manage plant diseases (Shahul Hameed et al., 2018). These bioactive compounds showed large peaks in the cell-free extracts of the bacterial strains indicating that they contribute significantly to antifungal and antibacterial activities (Soliman et al., 2022). We discovered that phenolic compounds had the greatest peak of all the secondary metabolites in the filtrates from endophytic bacteria cultures. As a result, structures formed from phenol are responsible for a wide range of bioactivities including antioxidant, cytotoxic, and antimicrobial (Negreiros de Carvalho et al., 2016). In accordance with Yu et al. (2002), *Pseudomonas and Bacillus* spp. produced a wide range of antibiotics including oligomycin, phenazine, pyoluteorin, pyrolnitrin, iturin, 2,4-diacetylphloroglucinol, zwittermycin A, pyocyanin, bacillomycin and surfactin that have antifungal properties. Shahul Hameed et al. (2018) mentioned that a set of 24 bioactive compounds were detected from *P. fluorescens* using GC-MS analysis. Additionally, such substances play an important role in many biological activities. Additionally, *E. cloacae* can secrete several antifungal metabolites which can inhibit many fungal pathogens (Montealegre et al., 2003).

This study also investigated the impact of Ec, PG, Pm, CF-Ec, CF-Pg, and CF-Pm on the severity of EB under both greenhouse and field conditions. There was a close correlation between results from the greenhouse and field studies. Ec and CF-Ec reduced EB severity compared to the other treatments. This may be due to endospheric microbes generating secondary active metabolites and exo-enzymes that protect plants from phytopathogens (Fouda et al., 2021). These microbes can also promote plant growth by producing phytohormones under biotic and abiotic stress. *Pseudomonas* spp. are known to produce broad-spectrum antibiotics which proved to be a major mechanism involved in their biocontrol activity against plant pathogens (Hemanandhini et al., 2019). Also, a certain *Enterobacter* spp. has been identified as plant growth enhancers since they have several growth-promoting activities (Ramesh et al., 2014; Selim et al., 2015). Basically, a symbiotic interaction is created between the bacteria and the plant once bacterial endophytes have colonized the tissues of the host. Biologically active metabolites are produced by bacterial endophytes, and the plant supplies nutrients to the bacterial colony (Hassan, 2017).

To better understand the mechanism of resistance, the expression β-1,3*-glucanase* gene was investigated in all EC-treated tomato plants in our study. This gene encodes one of the pathogenesis-related proteins (PR protein) that are included in several plant-pathogen interactions and associated with defense against plant diseases (Sharifi et al., 2012; Balbi-Peña et al., 2014). Plant *β*-*1*,3-glucanases play an important role in controlling the outcome of both symbiotic and antagonistic plant-microbe interactions by destroying non-self-glucan structures. Through the production of signaling glucans that activate global responses, plants can use these enzymes to hydrolyze-glucans present in the walls of microorganisms, helping to create a local defense barrier against germs (Perrot et al., 2022). In this study, Ec was the most effective treatment to reduce EB severity in tomato leaves infected by the three pathogenic fungi. So, it was worth investigating the effect of this bacteria on the expression of some antifungal genes such as the β-1,3-glucanases. The gene expression study focused on the Ec treatment only. Compared to the healthy and disease control, Ec significantly enhanced the expression of the β-1,3-glucanases gene. This increase was found to be associated with decreased disease severity. It was reported that higher expression of β-1,3-glucanases gene was correlated with lower disease severity in tomatoes and enhanced resistance to fungal diseases (Jongedijk et al., 1995). In our study, Ec increased the level of β-1,3-glucanases gene expression in tomato leaves infected by *C. lunata,* followed by *A. alternata* and *A. solani*. Ec also reduced symptom development of EB in tomato plants infected by *C. lunata,* followed by *A. alternata* and *A. solani*. Therefore, Ec delayed the development of EB symptoms of the pathogenic fungi providing sufficient protection against the disease.

To thoroughly investigate the effect of Ec on the expression of the β-1,3-glucanases gene, the tomato leaves infected with each fungal pathogen were collected after 3, 5, and 10 days. It was noted that the expression of the gene had a significant increase after 3 days from the inoculation with *C. lunata,* while it reached the maximum after 5 days from the inoculation with *A. alternata* and *A. solani*. This result could explain the low EB severity observed on tomato leaves infected by *C. lunata.* It suggests the Ec-induced an early expression of the β-1,3-glucanases gene, which reduced the symptoms on tomato compared to the other two fungal pathogens. Ec is a good biological agent which may have commercial value to reduce EB severity on tomato.

In conclusion, the study suggests that *E. cloacae* (Ec) and its culture filtrate, along with the other tested endophytic bacteria [*P. gessardii* (Pg) and *P. mediterranea* (Pm)], could serve as effective biocontrol agents against the growth of pathogenic fungi causing early blight in tomato plants. The presence of phenolic compounds and the activation of the plant’s defense responses contribute to the observed disease suppression effects. These findings hold potential for environmentally friendly strategies to manage early blight disease in tomato crops.

## Data availability statement

The original contributions presented in the study are included in the article/Supplementary material, further inquiries can be directed to the corresponding authors.

## Author contributions

NS designed the whole study and wrote the manuscript. H-AA and HK collected the data, analyzed the data, and helped in the genetic analyses. AE helped in the gene expression analysis. KA-E discussed the results and helped in editing the paper. ES discussed the results and edited the manuscript. AS conducted the gene expression and drafted the manuscript. All authors contributed to the article and approved the submitted version.

## Funding

Costs for open access publishing were partially funded by the Deutsche Forschungsgemeinschaft (DFG, German Research Foundation, grant 491250510).

## Conflict of interest

The authors declare that the research was conducted in the absence of any commercial or financial relationships that could be construed as a potential conflict of interest.

## Publisher’s note

All claims expressed in this article are solely those of the authors and do not necessarily represent those of their affiliated organizations, or those of the publisher, the editors and the reviewers. Any product that may be evaluated in this article, or claim that may be made by its manufacturer, is not guaranteed or endorsed by the publisher.
