# Supplementary material for: Exploring the mechanisms of endophytic bacteria for suppressing early blight disease in tomato (Solanum lycopersicum L.)
Source: Front Microbiol. 2023 Sep 21;14:1184343. doi: 10.3389/fmicb.2023.1184343 (PMC10551630; doi:10.3389/fmicb.2023.1184343)
Supplement: Supplementary file 1 [file Data_Sheet_1.zip › supplementary figures RIM R1.pptx]

## Slide 1
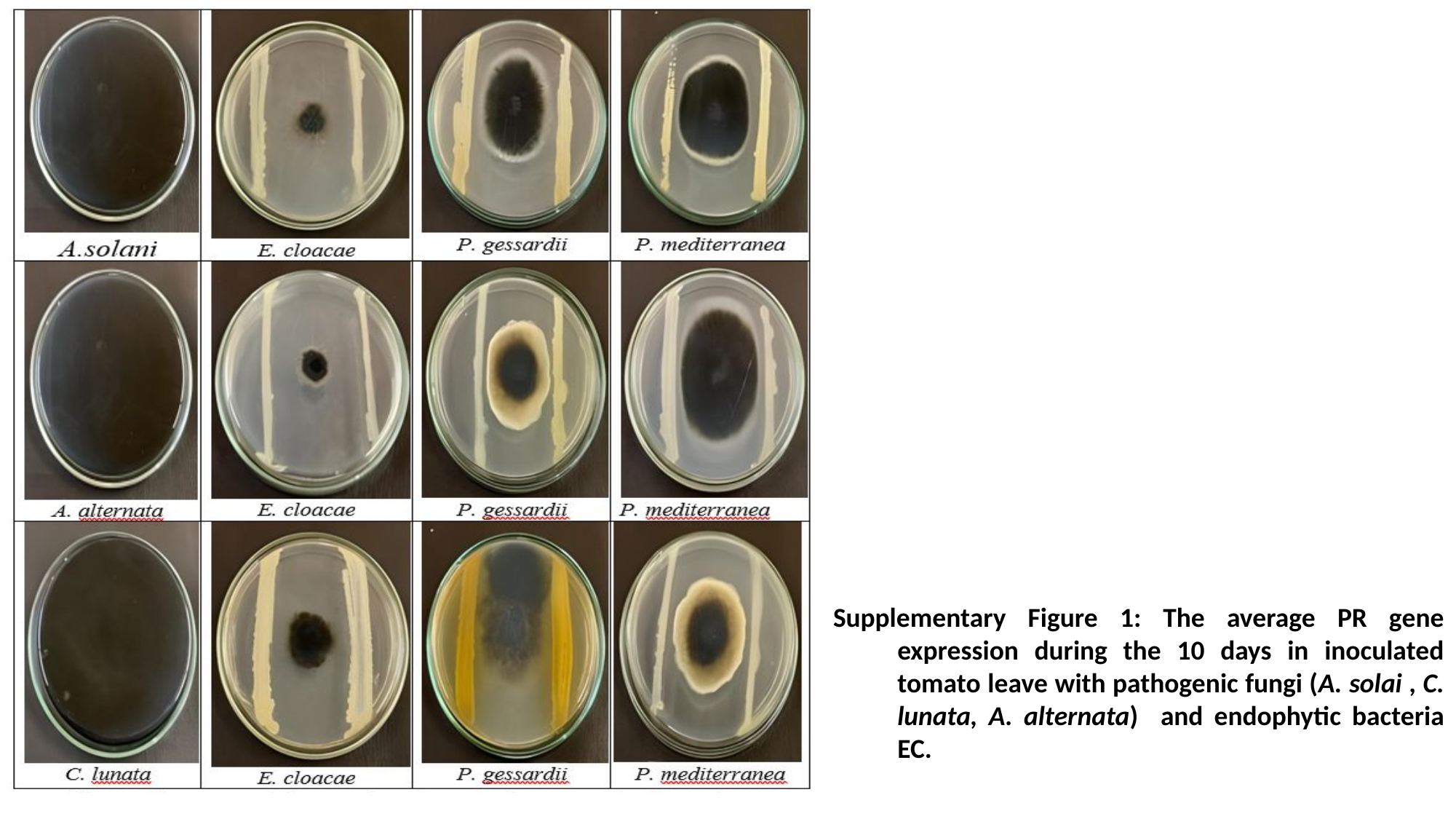

Supplementary Figure 1: The average PR gene expression during the 10 days in inoculated tomato leave with pathogenic fungi (A. solai , C. lunata, A. alternata) and endophytic bacteria EC.

## Slide 2
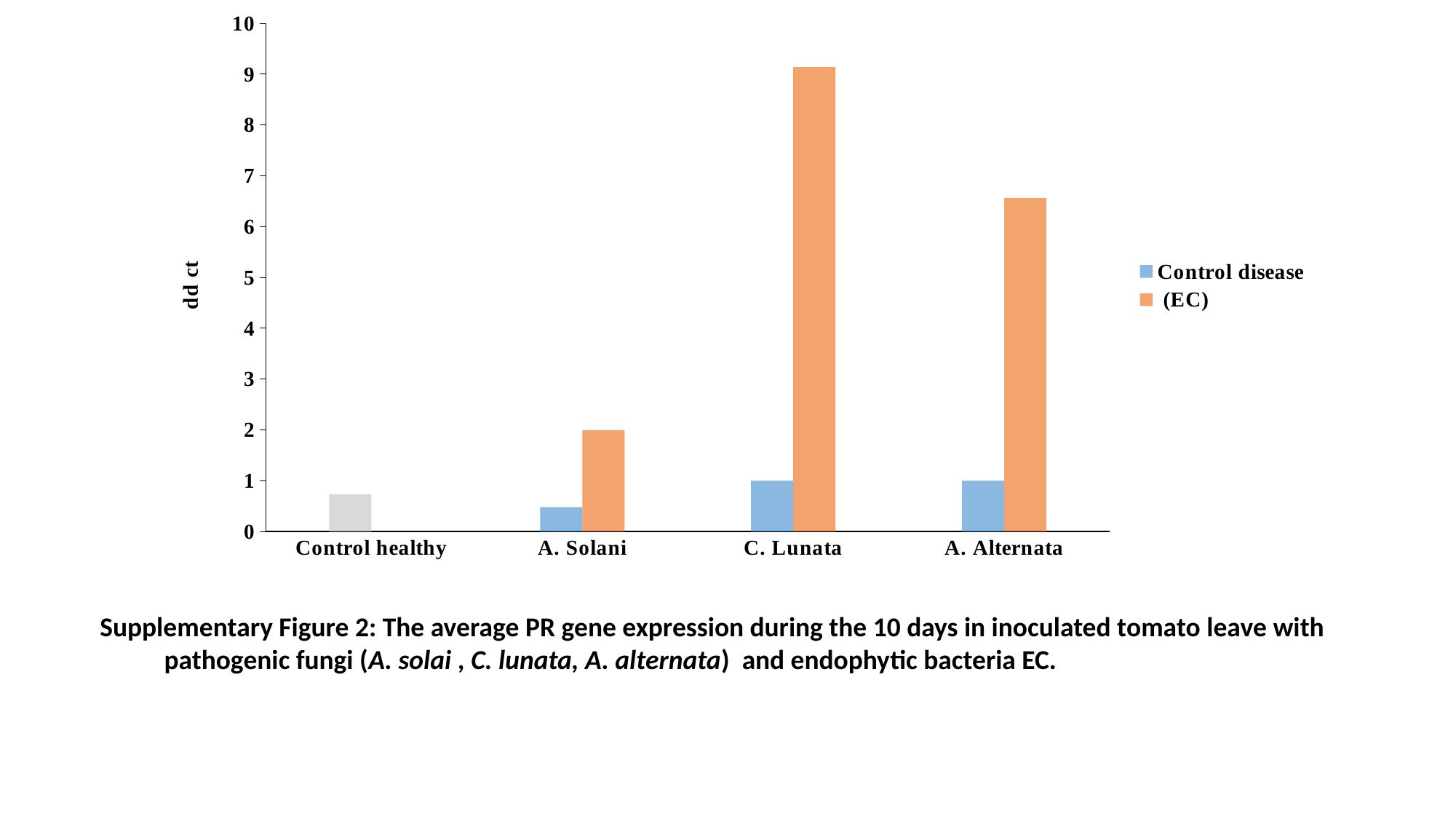

### Chart
| Category | | |
|---|---|---|
| Control healthy | 0.728525802601396 | None |
| A. Solani | 0.47798421273660047 | 1.9950030762855933 |
| C. Lunata | 0.9965402628278716 | 9.138637900822713 |
| A. Alternata | 0.996540262827868 | 6.566265535436034 |Supplementary Figure 2: The average PR gene expression during the 10 days in inoculated tomato leave with pathogenic fungi (A. solai , C. lunata, A. alternata) and endophytic bacteria EC.
