# Supplementary material for: Exploring the mechanisms of endophytic bacteria for suppressing early blight disease in tomato (Solanum lycopersicum L.)
Source: Front Microbiol. 2023 Sep 21;14:1184343. doi: 10.3389/fmicb.2023.1184343 (PMC10551630; doi:10.3389/fmicb.2023.1184343)
Supplement: Supplementary file 1 [file Data_Sheet_1.zip › supplementary Tables FIM R1.docx]

**Mechanism of endophytic bacteria to suppress tomato early blight disease**

Nashwa M. A. Sallam^1^*, Heba-Alla S. AbdElatah^1^, Hadeel M. M. Khalil Bagy^1^, Ameer Elfarash^4^, Kamal A. M. Abo‐ Elyousr^1,3^, Mohamed S. Mohamed^1^, Ahmed Sallam^2,4^*

^1^ Department of Plant Pathology, Faculty of Agriculture, Assiut University, 71526-Assiut, Egypt

^2^ Department of Genetics, Faculty of Agriculture, Assiut University, 71526-Assiut, Egypt

^3^ Department of Arid Land Agriculture, Faculty of Meteorology, Environment and Arid Land Agriculture, King Abdulaziz University, Jeddah 80208, Saudi Arabi

^4^ Department Genebank, Leibniz Institute of Plant Genetics and Crop Plant Research (IPK), Gatersleben, Germany

*corresponding author

Dr. Ahmed Sallam

[sallam@ipk-gatersleben.de](mailto:sallam@ipk-gatersleben.de) OR [amsallam@aun.edu.eg](mailto:amsallam@aun.edu.eg)

Prof. Dr. Nashwa Sallam

[nashwasallam@aun.edu.eg](mailto:nashwasallam@aun.edu.eg)

**Supplementary Table 1**: GC-MS analysis of CF-Ec

| **Retention time (RT)** | **Compound name** | **Molecular formula** | **Molecular weight** | **Area %** |
| --- | --- | --- | --- | --- |
| 3.18 | 2-Butanone, 3-hydroxy- (ç-Hydroxy-á-oxobutane) 22.60 | C4H_8_O_2_ | 88 | 22.60 |
| 3.34 | 1-Butanol, 3-methyl- (Isopentyl alcohol) | C_5_H_12_O | 88 | 0.26 |
| 3.49 | 2-Hexene, 2,3-dimethyl- | C_8_H_16_ | 112 | 0.24 |
| 3.76 | 2,3-Butanediol | C_4_H_10_O_2_ | 90 | 8.27 |
| 3.87 | 2,3-Butanediol | C_4_H_10_O_2_ | 90 | 8.60 |
| 5.44 | 1,3-Dioxan-5-ol, 4,4,5-trimethyl- | C_7_H_14_O_3_ | 146 | 0.30 |
| 7.05 | Hexanoic acid | C_6_H_12_O_2_ | 116 | 0.26 |
| 7.57 | Toluene, p-chloro- | C_7_H_7_Cl | 126 | 1.11 |
| 8.77 | 1-Methoxy-1,3,5-cycloheptatriene | C_8_H_10_O | 122 | 0.38 |
| 10.73 | Phenylethyl Alcohol | C_8_H_10_O | 122 | 1.07 |
| 11.90 | 7-HexadEcene | C_16_H_32_ | 224 | 0.91 |
| 12.42 | Naphthalene | C_10_H_8_ | 128 | 0.67 |
| 15.25 | 1-HexadEcene | C_16_H_32_ | 224 | 1.64 |
| 15.38 | n-TetradEcane | C_14_H_30_ | 198 | 0.35 |
| 16.10 | Methyl 9,10-dihydroxystearate | C_19_H_38_O_4_ | 330 | 0.60 |
| 17.37 | 6-Methyloctadecane | C_19_H_40_ | 268 | 0.22 |
| 17.92 | Phenol, 2,4-di-tert-butyl-Phenol, 2,4-bis(1,1-dimethylethyl)- | C_14_H_22_O | 206 | 45.27 |
| 19.74 | 1-Hexadecanol (n-Cetyl alcohol) | C_16_H_34_O | 242 | 2.13 |
| 19.92 | Tetradecane, 2,6,10-trimethyl- | C_17_H_36_ | 240 | 0.22 |
| 23.50 | Ethyl iso-allocholate | C_26_H_44_O_5_ | 436 | 0.25 |
| 24.92 | 9-Nonadecene | C_19_H_38_ | 266 | 2.14 |
| 27.12 | Phthalic acid, isobutyl octadecyl ester | C_30_H_50_O_4_ | 474 | 0.37 |
| 28.04 | Ethyl iso-allocholate | C_26_H_44_O_5_ | 436 | 0.29 |
| 29.24 | 1,2-Benzenedicarboxylic acid, butyloctyl ester | C_20_H_30_O_4_ | 334 | 0.46 |
| 29.53 | 1-Docosene | C_22_H_44_ | 308 | 1.40 |

**Supplementary Table 2**: GC-MS analysis of CF-Pg.

| **Retention time (RT)** | | | Compound name | **Molecular formula** | **Molecular weight** | **Area %** |
| --- | --- | --- | --- | --- | --- | --- |
| 3.48 | | 2,3-Dimethyl-2-hexene | | C_8_H_16_ | 112 | 1.10 |
| 3.71 | 3,5-Dimethyl-2-hexene | | | C_8_H_16_ | 112 | 7.32 |
| 3.93 | Hydrazinecarboxylicacid, phenylmethyl ester | | | C_8_H_10_N_2_O_2_ | 166 | 1.16 |
| 4.08 | Imidazole,2-amino-5-[(2-carboxy)vinyl]- | | | C_6_H_7_N_3_O_2_ | 153 | 1.10 |
| 7.58 | Toluene, p-chloro- | | | C_7_H_7_Cl | 126 | 4.82 |
| 8.79 | 1-Methoxy-1,3,5-cycloheptatriene | | | C_8_H_10_O | 122 | 1.53 |
| 11.91 | trans-3-DodEcene | | | C_12_H_24_ | 168 | 4.02 |
| 12.43 | Naphthalene))Albocarbon | | | C_10_H_8_ | 128 | 2.81 |
| 15.26 | 1-Hexadecanol (n-Cetyl alcohol) | | | C_16_H_34_O | 242 | 7.58 |
| 15.39 | Octadecane, 6-methyl - | | | C_19_H_40_ | 268 | 1.51 |
| 16.12 | 1,2,4-Trioxolane-2-octanoic acid,5-octyl-, methyl ester (Methyl oleateozonide) | | | C_19_H_36_O_5_ | 344 | 1.40 |
| 17.39 | Octadecane, 6-methyl- | | | C_19_H_40_ | 268 | 1.07 |
| 17.93 | Phenol, 2,4-bis(1,1-dimethylethyl)- or Phenol, 2,4-di-tert-butyl- | | | C_14_H_22_O | 206 | 34.49 |
| 19.75 | 1-Hexadecanol or n-Cetyl alcohol | | | C_16_H_34_O | 242 | 9.55 |
| 23.51 | cis-13-Eicosenoic acid | | | C_20_H_38_O_2_ | 310 | 1.48 |
| 24.93 | 1-NonadEcene | | | C_19_H_38_ | 266 | 8.92 |
| 27.13 | 9,12,15-OctadEcatrienoic acid,2-[(trimethylsilyl)oxy]-1-[[(trimethylsilyl)oxy]methyl]ethyl ester, (Z,Z,Z)- | | | C_27_H_52_O_4_Si_2_ | 496 | 1.19 |
| 28.05 | 9,10-SEcocholesta-5,7,10(19)-triene-3,24,25-triol, (3á,5Z,7E)- | | | C_27_H_44_O_3_ | 416 | 1.20 |
| 29.26 | 9,12,15-Octadecatrienoic acid,2-[(trimethylsilyl)oxy]-1-[[(trimethylsilyl)oxy]methyl]ethyl ester, (Z,Z,Z)- | | | C_27_H_52_O_4_Si_2_ | 496 | 1.84 |
| 29.54 | 17-Pentatriacontene | | | C_35_H_70_ | 490 | 5.93 |

**Supplementary Table 3:** GC-MS analysis of CF-Pm.

| **Retention time (RT)** | **Compound name** | **Molecule formula** | **Molecular weight** | **Area %** |
| --- | --- | --- | --- | --- |
| 3.47 | 3-Hexene, 2,2-dimethyl-, (Z)- | C_8_H_16_ | 112 | 1.06 |
| 3.70 | 2-Hexene, 3,5-dimethyl- | C_8_H_16_ | 112 | 7.03 |
| 3.92 | Hydrazin Ecarboxyli acid, phenylmethyl ester, or Carbobenzoxy hydrazide | C_8_H_10_N_2_O_2_ | 166 | 1.06 |
| 4.07 | Imidazole,2-amino-5-[(2-carboxy)vinyl]- | C_6_H_7_N_3_O_2_ | 153 | 1.15 |
| 7.57 | Benzene, 1-chloro-4-methyl- (Toluene, p-chloro) | C_7_H_7_Cl | 126 | 4.86 |
| 8.78 | 1-Methoxy-1,3,5-cycloheptatriene | C_8_H_10_O | 122 | 1.58 |
| 11.90 | trans-3-Dodecene | C_12_H_24_ | 168 | 4.00 |
| 12.42 | Naphthalene (Albocarbon) | C_10_H_8_ | 128 | 2.89 |
| 15.26 | 1-Hexadecanol (n-Cetyl alcohol) | C_16_H_34_O | 242 | 7.25 |
| 15.39 | Octadecane, 6-methyl- (6-Methyloctadecane) | C_19_H_40_ | 268 | 1.49 |
| 16.12 | 1,2,4-Trioxolane-2-, methyl ester (Methyl oleateozonide) | C_19_H_36_O_5_ | 344 | 1.41 |
| 17.92 | Phenol, 2,4-bis(1,1-dimethylethyl)-or Phenol, 2,4-di-tert-butyl- | C_14_H_22_O | 206 | 35.59 |
| 19.75 | 1-Hexadecanol (n-Cetyl alcohol) | C_16_H_34_O | 242 | 9.50 |
| 23.50 | 12-Methyl-E,E-2,13-octadEcadien-1-ol | C_19_H_36_O | 280 | 1.09 |
| 24.92 | 1-Docosene | C_22_H_44_ | 308 | 8.96 |
| 27.13 | 1,2-Benzenedicarboxylic acid, butyloctyl ester | C_20_H_30_O_4_ | 334 | 1.37 |
| 28.04 | 9,10-SEcocholesta-5,7,10(19)-triene-3,24,25-triol, (3á,5Z,7E)- (24,25-Dihydroxycholecalciferol) | C_27_H_44_O_3_ | 416 | 1.27 |
| 29.25 | 9,12,15-Octadecatrienoic acid,2-[(trimethylsilyl)oxy]-1-[[(trimethylsilyl)oxy]methyl]ethyl ester, (Z,Z,Z)- | C_27_H_52_O_4_Si_2_ | 496 | 1.80 |
| 29.54 | 5-OctadEcenal | C_18_H_34_O | 266 | 5.42 |
| 30.53 | 3-Pyridinecarboxylic acid,2,7,10-tris(acetyloxy)-1,1a,2,3,4,6,7,10,11,11a-dEcahydro-1,1,3,6,9-pentamethyl-4-oxo-4a,7a-epoxy-5H-cyclopenta[a]cyclopropa[f]cycloundEce-11ylester | C_32_H_39_NO_10_ | 597 | 1.23 |
